# Supplementary material for: Content-rich biological network constructed by mining PubMed abstracts
Source: BMC Bioinformatics. 2004 Oct 8;5:147. doi: 10.1186/1471-2105-5-147 (PMC528731; doi:10.1186/1471-2105-5-147)
Supplement: Additional File 2 — The original results of the above study (non-essential files are deleted to keep the file size under the limit set by BMC bioinformatics). [file 1471-2105-5-147-S2.bz2 › chilibotAdditionalFile2/dip05/678ID7876093E11085/html/TNFRSF1A_TRAP1.html]

 


 **TNFRSF1A** and **TRAP1** 
  
Found 10 abstracts in PubMed, retrieved 05.  
 

 What does Google say? 
 PDF only 
| .edu only 

---

**Interactive relationship** (e.g. stimulation, inhibition, etc)

**Neutral relationship**- We have used the yeast interaction trap  [ **TRAP1** ]  to isolate a protein, MADD, that associates with the death domain of TNFR1  [ **TNFRSF1A** ]  through its own C terminal death domain.  Ref: 9115275 J Biol Chem, 1997

**Non-interactive relationship** (e.g. studied together, co-existance, homology, etc.)

- By combined use of the yeast interaction trap  [ **TRAP1** ]  system and a peptide scanning library, the novel WD repeat protein FAN has been identified, which specifically binds to a cytoplasmic nine amino acid binding motif of TNF R55  [ **TNFRSF1A** ] .  Ref: 8808629 Cell, 1996
